# The 4AT, a rapid delirium detection tool for use in hospice inpatient units: Findings from a validation study

**DOI:** 10.1177/02692163241242648

**Published:** 2024-05-20

**Authors:** Elizabeth Arnold, Anne M Finucane, Stacey Taylor, Juliet A Spiller, Siobhan O’Rourke, Julie Spenceley, Emma Carduff, Zoë Tieges, Alasdair MJ MacLullich

**Affiliations:** 1 Marie Curie Hospice Edinburgh, Edinburgh, UK; 2 Clinical Psychology, University of Edinburgh, Edinburgh, UK; 3 Marie Curie Hospice Glasgow, Glasgow, UK; 4 Edinburgh Delirium Research Group, Ageing and Health, Usher Institute, University of Edinburgh, Royal Infirmary of Edinburgh, Edinburgh, UK; 5 Department of Computing, School of Computing, Engineering and Built Environment, Glasgow Caledonian University, Scotland, UK

**Keywords:** Delirium, assessment, palliative, hospice, validation, hospice inpatient, 4 ‘A’s test, 4AT, detection, terminally ill

## Abstract

**Background:**

Delirium is a serious neuropsychiatric syndrome with adverse outcomes, which is common but often undiagnosed in terminally ill people. The 4 ‘A’s test or 4AT (www.the4AT.com), a brief delirium detection tool, is widely used in general settings, but validation studies in terminally ill people are lacking.

**Aim:**

To determine the diagnostic accuracy of the 4AT in detecting delirium in terminally ill people, who are hospice inpatients.

**Design:**

A diagnostic test accuracy study in which participants underwent the 4AT and a reference standard based on the fifth edition of the Diagnostic and Statistical Manual of Mental Disorders. The reference standard was informed by Delirium Rating Scale Revised-98 and tests assessing arousal and attention. Assessments were conducted in random order by pairs of independent raters, blinded to the results of the other assessment.

**Setting/participants:**

Two hospice inpatient units in Scotland, UK. Participants were 148 hospice inpatients aged ⩾18 years.

**Results:**

A total of 137 participants completed both assessments. Three participants had an indeterminate reference standard diagnosis and were excluded, yielding a final sample of 134. Mean age was 70.3 (SD = 10.6) years. About 33% (44/134) had reference standard delirium. The 4AT had a sensitivity of 89% (95% CI 79%–98%) and a specificity of 94% (95% CI 90%–99%). The area under the receiver operating characteristic curve was 0.97 (95% CI 0.94–1).

**Conclusion:**

The results of this validation study support use of the 4AT as a delirium detection tool in hospice inpatients, and add to the literature evaluating methods of delirium detection in palliative care settings.

**Trial registry:**

ISCRTN 97417474.

**What is already known about this topic?**

- Delirium is a serious neuropsychiatric syndrome with adverse outcomes, which is common but often undiagnosed in terminally ill people.
- The 4AT is a brief delirium detection tool, which is widely used in general settings, but validation studies in the terminally ill are lacking.

**What this paper adds?**

- This study adds to the small literature evaluating methods of delirium detection in terminally ill people and is the first validation study of the 4AT in a hospice inpatient setting.
- The 4AT had a sensitivity of 89% and specificity of 94% for delirium detection, as assessed by the DSM-5 delirium reference standard, in this study of terminally ill people in hospice inpatient units.
- These findings support the use of the 4AT as a validated delirium detection tool in hospice inpatient settings.

**Implications for practice, theory or policy**

- The simplicity and brevity of the 4AT are advantages in clinical practice.
- Implementation studies are essential to support routine delirium assessment of terminally ill people.
- Further research evaluating 4AT use in community settings is needed, specifically homes and care homes, where delirium assessment presents different challenges.

## Background

Delirium is a serious and distressing neuropsychiatric condition,^1^ which is common in terminally ill people. Recent studies suggest delirium is present in over a quarter of patients admitted to hospices, and that prevalence increases towards the end of life.^2^ Delirium often remains undiagnosed and hence under-treated.^3^ The hypoactive subtype is common amongst terminally ill people, ^2^ yet this subtype is more likely to go underdiagnosed,^4,5^ perhaps due to overlapping symptoms with depression, dementia and fatigue.^4,6^

Delirium may be reversible, but its development may also signal the person is approaching end of life.^7^ Earlier detection facilitates more timely management, which may result in better patient outcomes.^8^ Palliative and generic delirium guidance recommends routine delirium screening, including detection tool use, on admission to hospitals and other care settings or if delirium is suspected.^9–14^ A UK survey in 2019 reported that only a third of palliative medicine specialists used delirium screening tools, with more relying on clinical judgement alone.^15^ Yet there is increasing awareness that clinical judgement alone risks delirium going under-diagnosed.^5,16^

Many delirium detection tools are available, with some designed to detect delirium at first assessment and when delirium is suspected. Other tools monitor for new onset delirium in inpatients, measure delirium severity or are primarily used for research purposes.^8^ A systematic review and meta-analysis in 2021 reported the characteristics and performance of 14 delirium detection tools used in palliative care.^17^ These were the Bedside Confusion Scale, the Communication Capacity Scale, Clinical Assessment of Confusion (versions A and B), the Confusion Assessment Method (CAM) and brief Confusion Assessment Method (bCAM), the Delirium Observation Screening Scale (DOS), the Delirium Rating Scale (DRS) and the revised version (DRS-R-98), the Memorial Delirium Assessment Scale (MDAS), the Single Question in Delirium (SQiD), the Neelon and Champagne Confusion Scale (NEECHAM), the Nursing Delirium Screening Scale (Nu-DESC) and the Visual Analogue Scale for Acute Confusion (VASAC). Of the 14 tools examined, 12 were assessed in only one study. Sample sizes ranged from 19 to 2343, though most studies had relatively small samples. The review concluded it was difficult to make recommendations given the heterogeneity of the studies, and that choice of tool may depend on the population, setting and expertise of healthcare professionals. The CAM and the MDAS were proposed for assessing delirium amongst patients able to co-operate with cognitive testing. Alternatively, the DOS and Nu-DESC, which are solely dependent on the assessor’s observations, were deemed suitable for non-verbal patients approaching the end of life.^17^ A subsequent study, not included in the 2021 review, compared the performance of the SQiD and short CAM to psychiatrist interview amongst oncology patients – this reported high levels of specificity (87% and 100% respectively), but lower sensitivity levels (44% and 26%).^18^ The challenges of providing adequate training to use the CAM were acknowledged, as this and previous studies have reported accuracy may be dependent on user expertise.^19,20^

Despite the range of delirium detection tools assessed, the 4AT (www.the4AT.com) remains unvalidated in palliative care populations. Whilst the CAM is recommended for use by American and Canadian delirium guidance,^13,21^ the 4AT is recommended as the main delirium assessment tool in non-ICU settings by the UK National Institute of Health and Care Excellence (NICE) and the Scottish Intercollegiate Guidelines Network (SIGN),^10,11^ as well as Australian guidance.^12^

**Figure 1.**
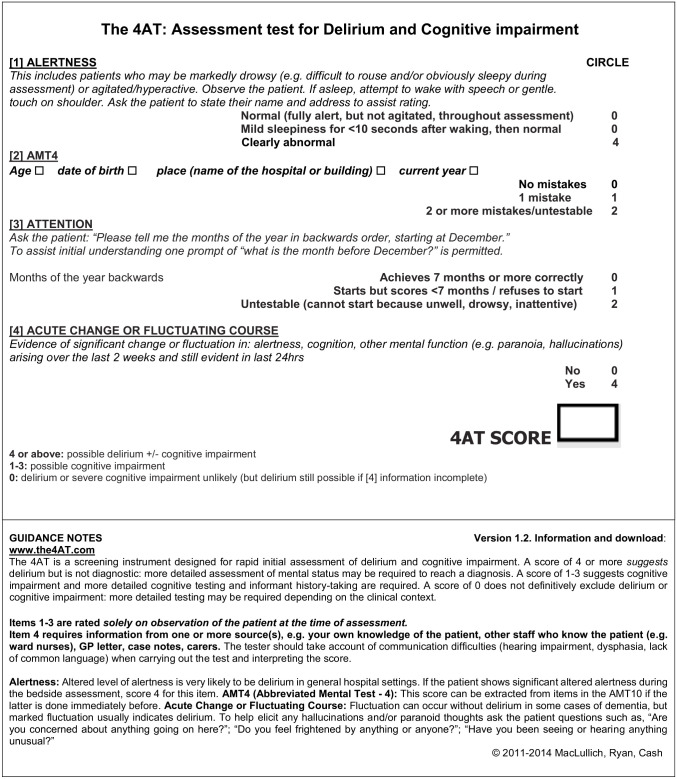
The 4AT.^22^

The 4AT is a short bedside test widely used by healthcare professionals in routine clinical practice to determine if patients may have delirium^22^ (Figure 1). It incorporates four items (the 4 ‘A’s) to score from 0 to 12, including an observational measure of **A**lertness, the **A**bbreviated Mental Test-4 and **A**ttention score (participant asked to say the months of the year in backwards order), and evidence of **A**cute change or fluctuation in alertness, cognition or mental function arising over the preceding 2 weeks and still evident in the last 24-hour. The 4AT has some advantages over other detection tools, in that it requires little training prior to use, is simple and quick to administer and can be used with non-verbal patients, who are either very agitated or drowsy. Whilst unvalidated in palliative care populations, the 4AT has been extensively validated in other populations, with 25 studies involving over 5000 patients.^22^ A systematic review and meta-analysis of 17 studies of elderly adults (⩾65 years) published in 2021 reported pooled sensitivity of 88% (95% CI 80%–93%) and specificity of 88% (95% CI 82%–92%).^23^

## Aim

The aim of the study was to determine the accuracy of the 4AT in detecting delirium amongst terminally ill people, in hospice inpatient settings, in terms of sensitivity and specificity.

## Methods

### Design

**Figure 2.**
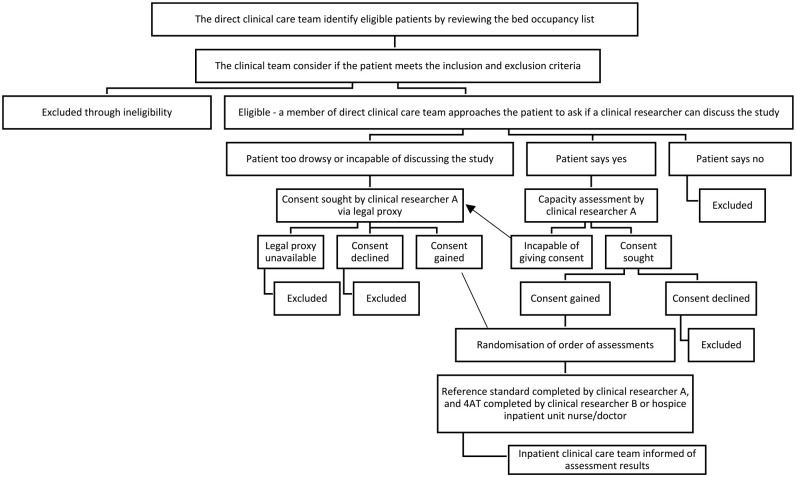
Study overview flow chart – this chart has been adapted from Shenkin et al.^25^ (http://creativecommons.org/licenses/by/4.0/).

We conducted a test validation study comparing the accuracy of the 4AT against a reference standard assessment, based on the diagnostic criteria of the fifth edition of the Diagnostic and Statistical Manual of Mental Disorders (DSM-5)^1^ (Figure 2). The study was registered on the ISCRTN (ISRCTN97417474 – 21/2/20), and full methodology is available in the protocol paper.^24^

### Sites

The sites were two hospice inpatient units in Scotland, UK. Approval was gained for a third site, which later withdrew from the study due to lack of resources to undertake data collection. In the UK, hospices are part of specialist palliative care services, which provide care to terminally ill people with complex needs, that cannot be met by generalist services.

### Population

#### Sample size and recruitment

Based on a sensitivity and specificity of 0.85 and a minimal acceptable lower confidence level of 0.75, we estimated a sample size of 176 participants would be required to complete the study assessments.^26^ To achieve this, and given the likelihood of withdrawals, we sought to recruit approximately 240 participants.^24^

Recruitment began in October 2019 but halted at the start of the COVID-19 pandemic in March 2020, before restarting between July 2021 and April 2022. People admitted to the hospice inpatient units were eligible to participate, and the study inclusion and exclusion criteria are shown in Table 1. Those who fulfilled the eligibility criteria, were approached by their direct clinical team, which may have included the clinical researchers (hospice nurses SO, ST and doctors JSp, EA). Eligible patients, who were interested, received verbal and written information about the study from researchers. People deemed to have capacity to consent by the researchers were invited to sign the consent form. If the researcher assessed the person as lacking capacity to consent, their legal proxy (Welfare Attorney, Guardian or nearest relative, approached in that order) was asked on their behalf, as permitted by the Adults with Incapacity (Scotland) Act 2000.^27^ The clinical researchers had allocated research time, but the study was also conducted alongside their usual clinical practice, which influenced the timing of the initial approach and recruitment.

**Table 1.** Inclusion and exclusion criteria.

**Inclusion criteria**
Aged 18 years or over
Admitted acutely to the hospice inpatient unit
**Exclusion criteria**
Acute life-threatening illness requiring time critical intervention (e.g. suspected spinal cord compression)
High level of patient and family distress, as judged by the clinical team
Severe dysphasia
Combined hearing and visual impairment which would limit participation in the study’s tests
Being unable to communicate in English
Coma

### Assessments

Participants underwent the 4AT and a reference standard assessment within a 3-hour period. Assessments were conducted in random order by pairs of independent assessors, who were blinded to the results of the other assessment. Unblinding occurred immediately following completion of both assessments. Participants’ involvement in the study was solely for the duration of the two assessments, within the 3-hour time period.

### 4AT assessment

The 4AT was completed by a clinical researcher or a hospice inpatient unit nurse or doctor. All had received generic delirium education (undergraduate and/or as continuing professional development) prior to the study – however only the clinical researchers completed study-specific delirium assessment training.^[Bibr bibr22-02692163241242648]^ The 4AT incorporates four items to score from 0 to 12 (Figure 1).^22^ For the purposes of this study, a score of more than 3 was considered ‘delirium present’, whereas scores of 0–3 were designated ‘delirium absent’.^28^

### Reference standard assessment

The reference standard assessment was completed by the clinical researcher, who had completed the consent process with the participant. The reference standard was centred on the delirium diagnostic criteria in the fifth edition of the Diagnostic and Statistical Manual of Mental Disorders (DSM-5)^1^ and described in more detail in the protocol paper.^24^ The battery of tests assessing attention and cognition were supplemented by collateral history from family members, carers or other staff members (other than the 4AT assessor; Supplemental File 1). Following the reference standard assessment, the participant was allocated to one of four categories: ‘delirium present’, ‘delirium absent’, ‘possible delirium’ (some DSM-5 delirium criteria were positive, but not all, due to missing information) or ‘undetermined’ (some, but not all, DSM-5 delirium criteria were positive).

The reference standard assessors discussed all ‘possible delirium’ or ‘undetermined’ cases with an expert panel (JAS, ZT and AMJM), as well as other cases where there was uncertainty regarding final group allocation. The expert panellists were blinded to the initial assessment outcomes, until this final allocation was complete.

### Data collection

The following data were collected for each participant: age, sex, primary diagnosis (including cancer type if relevant), Australia-modified Karnofsky Performance Status (AKPS) scale, presence of dementia or learning disability, medication use (specifically opioids, antipsychotics and benzodiazepines), outcome of hospice admission (discharge or death).

### Outcomes

We reported the sensitivity and specificity of the 4AT compared to the reference standard delirium assessment for individual and combined sites. Sensitivity was defined as the proportion accurately identified as having delirium, and specificity as the proportion accurately identified as being without delirium. We also reported the area under the receiver operating characteristic curve (AUROC) and its 95% confidence interval. The AUROC is a measure of the performance of the test, ranging between 0 and 1, with a higher score indicating a more accurate test.^29^ Further secondary analyses were completed whereby the indeterminate cases (‘possible delirium’ and ‘undetermined’), pre- and post-expert panel, were either assumed to have delirium or not have delirium. IBM SPSS version 27 was used to support the analysis.

## Results

**Figure 3.**
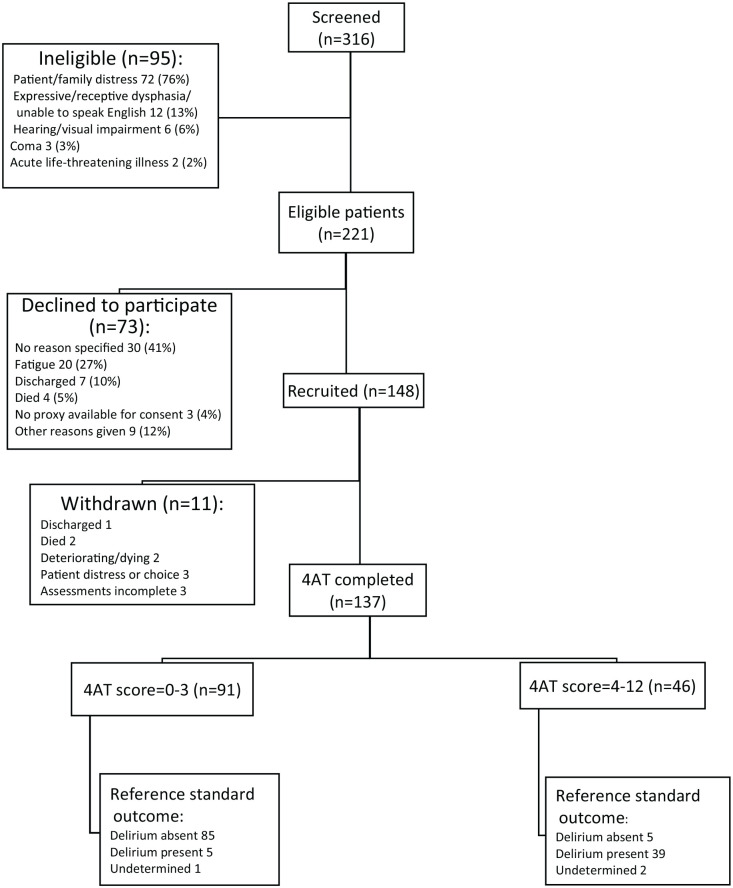
Standards for Reporting Diagnostic Accuracy diagram (STARD) showing summary of eligibility, participation and outcomes, final reference standard delirium status. During the study, it became apparent a proportion of ineligible patients had been inaccurately allocated to the category ‘coma’, when the reason was more appropriately described as patient and/or family distress associated with the dying phase. Where there was sufficient information, these cases were retrospectively reallocated from coma to ‘patients and/or family distress’. The proportion of reasons given for exclusion were affected, but not the analysis of the main results.

Three hundred and sixteen patients were screened to participate in the study, with over two-thirds (70%, 221/316) being eligible to participate (Figure 3). Of the 221 patients eligible to participate, 67% (148/221) were recruited, with 137 (93%) completing both the assessments. Eleven withdrew between recruitment and data collection (Figure 3).

Sixteen (12%) of the 137 participants, who completed the assessments, were discussed with the expert panel – 12 of these cases (12/16) originally had a ‘possible delirium’ or ‘undetermined’ diagnosis. Following the expert panel, 134/137 participants (98%) had a definitive diagnosis of either ‘delirium present’ or ‘delirium absent’. The three participants with ‘undetermined’ diagnoses were excluded from the primary analysis, yielding a final sample of 134.

### Characteristics of participants

The mean age was 70.3 (SD = 10.6) years and 50% (67/134) were female. Table 2 shows the clinical and demographic features of the 134 participants, who completed the assessments, both as a whole group and as subgroups- delirium present or delirium absent determined by their reference standard delirium final status. The median and mode Australia-modified Karnofsky Performance Status (AKPS) score was 50% – this score reflects patients in need of considerable assistance and frequent medical care.^30^

**Table 2.** Clinical and demographic characteristics of participants by final reference standard delirium status (*n* = 134).

	All participants *n* = 134	Delirium absent *n* = 90	Delirium present *n* = 44
Age in years: Mean (SD)	70.3 (10.6)	68.7 (10.6)	73.5 (10.0)
Cancer diagnosis (*n* = 132)^a^	124 (94%)	85 (94%)	39 (89%)
Gender			
Female	67 (50%)	44 (49%)	23 (52%)
Male	67 (50%)	46 (51%)	21 (48%)
Australia-Modified Karnofsky Performance Status scale % (*n* = 131)^b^: median (mode)	50% (50%)	50% (50%)	40% (30%)
Participant using medication <24 h of delirium assessments			
Opioids	122 (91%)	85 (94%)	37 (84%)
Antipsychotics^c^	43 (32%)	17 (19%)	26 (59%)
Benzodiazepines	71 (53%)	48 (53%)	23 (52%)
Participant outcome			
Discharge	47 (35%)	39 (43%)	8 (18%)
Death	69 (51%)	36 (40%)	33 (75%)
Unknown	18 (13%)	15 (17%)	3 (7%)

a data missing for 2 participants.

b data missing for 3 participants.

c indication for use was unspecified - may have been as antiemetic or to palliate agitated delirium symptoms.

Most participants (94%, 124/132) had a primary diagnosis of malignancy, with only 6% (8/132) having non-malignant disease. Of those whose cancer type was specified (85%, 105/124), approximately a third had lung cancer (34%), another third had cancer of gastrointestinal origin (32%), 8% had prostate cancer, 7% gynaecological, 6% breast and 5% urological cancers. The prevalence of dementia was 6% (8/134) as documented in their medical case notes or from informant history, with an additional 2% (3/134) of participants having a history of cognitive impairment ranging between months to 2 years. The reason for hospice admission was symptom control and/or end of life care for 88% and end of life care alone for 10%. At least half of participants (52%) died during their hospice admission.

### Diagnostic test accuracy of the 4AT

#### Primary analysis

**Figure 4.**
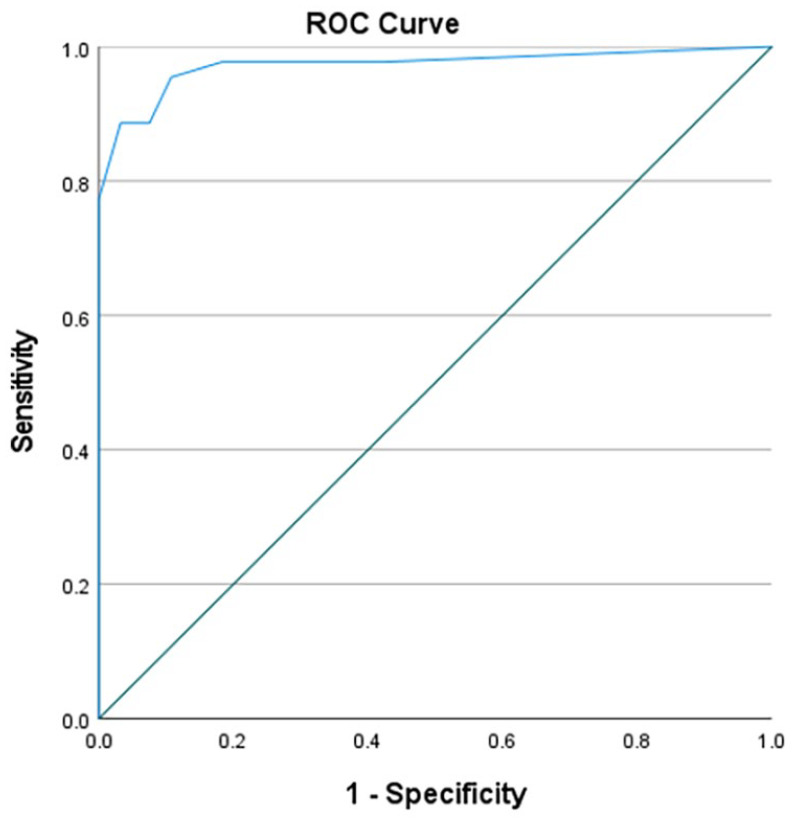
Receiver operating characteristic curve for 4AT diagnostic accuracy.

The sensitivity of the 4AT using the standard cut-off score of >3 was 89% (95% CI 79%–98%) and the specificity was 94% (95% CI 90%–99%). The area under the receiver operating characteristic curve was 0.97(95% CI 94%–100%; Figure 4).

#### Subgroup analysis for the two sites

4AT sensitivity was 88% and specificity 92% at hospice site B (*n* = 86) and 90% and 97% respectively at hospice site A (*n* = 48; Table 3).

**Table 3.** Results of assessments for individual sites: Site A (*n* = 48) and Site B (*n* = 86).

	Site A: Reference standard assessment		Site B: Reference standard assessment	
	Delirium absent	Delirium present	Delirium absent	Delirium present
4AT outcome				
Score 0-3: delirium absent	37	1	48	4
Score 4-12: delirium present	1	9	4	30
Total	38	10	52	34
Sensitivity (%)	90		88	
Specificity (%)	97		92	

#### Secondary analyses

##### Pre-expert panel

Table 4 shows the assessment results, pre-expert panel. Using the reference standard assessors’ initial DSM-5 outcomes, ‘delirium present’/‘delirium absent’, (excluding 12 cases of ‘undetermined’ and ‘possible delirium’), the 4AT sensitivity score was 93% and specificity 95%. Alternatively, inclusion of these 12 cases, assuming delirium was present for all 12 cases, sensitivity was lower at 79%, but the specificity result was similar at 95%, compared to the primary analysis. If delirium was assumed to be absent for these 12 cases, the sensitivity of the 4AT was 93% and specificity 92%.

**Table 4.** Results of pre-expert panel outcomes for the reference standard assessment.

	Reference standard assessment, pre-expert panel		
	Delirium absent	Delirium present	Possible delirium or undetermined
4AT outcome			
Score 0–3: delirium absent	80	3	8
Score 4–12: delirium present	4	38	4
Total	84	41	12

##### Post-expert panel

Assuming the 3 ‘undetermined’ cases excluded from the primary analysis had delirium, resulted in sensitivity of 87% and specificity of 94% for the 137 participants. Conversely if the three ‘undetermined’ cases did not have delirium, resulted in overall sensitivity of 89%, and a specificity of 92%.

## Discussion

We found that the 4AT had a sensitivity of 89% and specificity of 94% for delirium detection, as assessed independently by the DSM-5 delirium reference standard assessment in hospice inpatients. The area under the receiver operating characteristic curve was high at 0.97. Our findings indicate that the 4AT is a valid tool for detecting delirium in hospice inpatients.

The sensitivity of the 4AT for delirium detection in this hospice study is comparable to that reported in the 2021 systematic review and meta-analysis of 17 studies in older adults (aged ⩾65 years), with a reported pooled sensitivity of 88%.^3^ The specificity result here was higher (94% versus 88%), which may have been due to the hospice population being younger (28% of participants were under the age of 65 years), more homogeneous in terms of their primary diagnosis (94% had a cancer diagnosis), with lower prevalence of dementia (6%). Studies included in the 2021 systematic review of older adults ^23^ reported lower specificity scores for the 4AT amongst populations with higher prevalence of dementia.^28,31,32^

The sensitivity and specificity results of the 4AT from this hospice study are also comparable with studies of other delirium detection tools in palliative populations, including the Confusion Assessment Method and its shorter variants, the Memorial Delirium Assessment Scale and Delirium Observation Screening Scale.^17,18^

### Strengths and weaknesses

This study had several strengths. We used a comprehensive process to inform the reference standard assessments, which improves replicability and transparency. The prevalence of delirium in this study as detected by the reference standards was 33%, which suggests the sample is reflective of hospice inpatient units described elsewhere in the literature.^2^ Reference standard and index (4AT) assessments were conducted independently. Indeterminate cases were managed using an explicit process with an expert panel blinded to 4AT scores. A further strength of the study was that it was conducted across two sites, with assessments completed by nurses and doctors working within the units, which may have supported the relatively high levels of recruitment and assessment completion. Despite Covid pandemic restrictions, limited visiting continued, thus permitting relatively easy access to family members, who provided collateral history to support the accuracy of delirium assessments. The secondary analyses were also supportive of the primary analysis – the sensitivity and specificity results were higher for site A (90% and 97% respectively) compared to site B (88% and 92% respectively), although the prevalence of delirium was lower at site B (21%) compared to site A (39%). The variation in delirium prevalence between sites is unclear, but may have been due to differences in timing of assessments following admission, although we do not have the data to support this.

Limitations of this study should be acknowledged. Most participants had cancer as their primary diagnosis, so results may not be generalisable to those with advanced non-malignant disease or more heterogenous palliative populations. Furthermore, although the 4AT can be used to assess patients unable to communicate, the study’s eligibility criteria excluded terminally ill people unable to speak English or with severe dysphasia according to their primary diagnosis. Hence some with cerebral malignancies or neurological diseases, who may be more challenging to assess for delirium, will have been excluded from participation. Related to this point, ‘patient and/or family distress’ was the most frequent exclusion criterion, particularly for those entering the very terminal phase of their illness (last days or week of life). Previous studies have shown that the prevalence of delirium increases towards the end of life,^2^ yet delirium detection tool use may be more complex during the dying phase of the patient’s illness.

Recruitment bias may have been present, with those at moderate risk of delirium potentially being more challenging to recruit, compared to those at low or high risk. That is, those at moderate risk may have been more readily viewed as ineligible due to distress or other issues. In contrast, those at low risk of delirium may have been more easily judged as eligible due to having capacity, while those at high risk of delirium could be approached for consent via their legal proxy. Spectrum bias in delirium studies is an inherent challenge in studies requiring patient or proxy consent. Lower 4AT completion rates may be found in routine clinical practice. Use of the 4AT, including completion rates and scores across the severity spectrum, should be further evaluated using observational data from clinical practice. The target recruitment rate of 240 participants was not achieved despite the extended recruitment period, however the numbers completing the study remain comparable with other delirium detection tool validation studies in hospice or palliative inpatient populations.^17^

### Implications for practice

Our findings support the use of the 4AT for delirium detection in hospice inpatient settings. The simplicity and brevity of the 4AT, and functionality to assess non-verbal patients are advantages in clinical practice. Delirium prevalence increases during patients’ hospice stay, so it would seem appropriate to consider further 4AT use during the patient’s admission, whenever delirium is suspected.^2,33^ Whilst the 4AT does not require special training prior to use, adequate knowledge about the condition delirium is important in detection tool use,^34^ thus staff training in understanding delirium, its detection and management is recommended

### Further research

Implementation studies are essential to support routine delirium assessment for terminally ill people in clinical practice. Barriers to adoption of the 4AT and other tools have been described in other settings^35^ and need to be examined and addressed regarding terminally ill populations. This could be achieved, by examining completion rates and positive score rates in routine practice, as has been done in other settings.^36^ Further research evaluating 4AT use amongst terminally ill people in community settings is needed, specifically home and care homes, where delirium assessment presents different challenges.^37^

## Conclusion

The 4AT is a short delirium detection tool that can be used to identify delirium in terminally ill people receiving palliative care in hospice inpatient settings. Embedding the 4AT within standard clinical assessment is recommended.

## Supplemental Material

sj-pdf-1-pmj-10.1177_02692163241242648 – Supplemental material for The 4AT, a rapid delirium detection tool for use in hospice inpatient units: Findings from a validation study Supplemental material, sj-pdf-1-pmj-10.1177_02692163241242648 for The 4AT, a rapid delirium detection tool for use in hospice inpatient units: Findings from a validation study by Elizabeth Arnold, Anne M Finucane, Stacey Taylor, Juliet A Spiller, Siobhan O’Rourke, Julie Spenceley, Emma Carduff, Zoë Tieges and Alasdair MJ MacLullich in Palliative Medicine
