# Supplementary material for: The 4AT, a rapid delirium detection tool for use in hospice inpatient units: Findings from a validation study
Source: Palliat Med. 2024 May 20;38(5):535–45. doi: 10.1177/02692163241242648 (PMC11170929; doi:10.1177/02692163241242648)
Supplement: sj-pdf-1-pmj-10.1177_02692163241242648 – Supplemental material for The 4AT, a rapid delirium detection tool for use in hospice inpatient units: Findings from a validation study [file sj-pdf-1-pmj-10.1177_02692163241242648.pdf]

Participant ID – 3 digit no	Site	Date of visit
_______________	A ☐ B ☐ C ☐	____/___/____

Case Report Form – Reference Assessment

SCREENING

EXCLUSION CRITERIA

ELIGILITY AND INCLUSION OF THE PATIENT IN THE STUDY HAS BEEN DISCUSSED WITH THE PATIENT'S DIRECT CLINICAL CARE TEAM		
1. Acute life threatening illness requiring time critical intervention	Yes ☐	No ☐
2. Coma	Yes ☐	No ☐
3. Unable to speak English?	Yes ☐	No ☐
4. Severe dysphasia	Yes ☐	No ☐
5. Combined severe hearing and visual impairment, which would limit participation in the study's tests	Yes ☐	No ☐
6. High level of patient and family distress, as judged by the clinical team	Yes ☐	No ☐
If column complete then proceed with assessment		

Does the patient have capacity to provide consent or a suitable relative who can consent on the patient's behalf?	Yes ☐	No ☐	Not known
Is the patient participating in any other research? (this may not exclude the patient from participating in this study)	Yes ☐	No ☐	Not known
Has the patient deteriorated since first identified?	No ☐	Yes ☐	

Eligibility Screening Completed: Yes ☐ No ☐

**Researcher name**	**Researcher signature**
**Date** ____/____/____	

CONSENT

Patient has capacity to consent	Yes ☐	No ☐
As patient lacks capacity, suitable legal proxy has been asked	Yes ☐	No ☐
Patient /legal proxy has consented – this consent remains valid	No ☐	Yes ☐

**Name of Researcher performing the reference standard assessment**	**Researcher signature**
**Date of assessment** _____/_____/______	

Participant ID – 3 digit no	Site	Date of visit
_______________	A ☐ B ☐ C ☐	___/___/___

To be completed by Researcher (reference assessor)

Assessment Details

Start time: _______ Finish time: ________

Hearing loop required: Yes / No

Please do the interview questions and tests in the order given below, however use additional questions and conversation as required to help inform the DRS-98 and other items, and to provide reassurance etc.

Participant interview: brief guidance notes

The initial introductions and discussion with the patient have the aims of:
a.) Maintaining rapport and helping the patient feel comfortable with the interview and testing process, and
b.) Providing information about any abnormalities in mental status as per the DRS-R98.

During the whole interaction (discussion and testing), as well as recording information on the tests themselves, the DRS-R98 manual states the tester should be ‘examining and observing the patient throughout the interview for many features such as language, thinking, movements, paying attention, emotional control, and so on’.

Take notes as required regarding any abnormalities of mental state, changes in level of arousal/attention/affect, apparent hallucinations or delusions etc. These observations will go together with your direct questions to the participant, the casenotes review, discussions with staff or professional carers, and other sources of information, to inform the items on the DRS-R98 and the DSM-V criteria.

Participant interview: process

Start by introducing yourself.
Check they are feeling okay (too cold/warm, if they would like a drink – check they are safe to drink, before asking this).
Consider asking them why they are in the hospice.
Then briefly remind the participant of what you will be asking them to do – explain that you are going to go through a small number of different shorts tests of concentration and memory. Then proceed with the testing.

Participant ID – 3 digit no	Site	Date of visit
______________	A ☐ B ☐ C ☐	____/___/____

Orientation and memory

*** PRIORITY*** Please ask the participant the following questions. **UTA ☐**

	Correct	Incorrect	No response	UTA	Participant response
What is your age?					
What is your date of birth?					
What city are we in just now?					
What building are we in just now?					
What is the month?					
What is the year?					
What is the time of day (within 2 hours) without looking at your watch/clock					

Quick test of short-term memory (Subtest of the DRS-R-98)

PRIORITY
Show the participant the three images and say aloud "*Please look at these pictures. This is a lemon, a key and a ball*".
Then remove the images and ask the participant *"Could you now tell me again, what pictures you have just seen"*.
After participant repeats, say *"Try to remember them because I am going to ask you about this later"*.
Score only the first trial (but repeat 3 times if necessary).

Correctly identified: Lemon: Yes ☐ No ☐ UTA ☐
Key: Yes ☐ No ☐ UTA ☐
Ball: Yes ☐ No ☐ UTA ☐
Number of trials required: 1 ☐ 2 ☐ 3 ☐

Other reponses

Participant ID – 3 digit no	Site	Date of visit
_______________	A ☐ B ☐ C ☐	____/___/_____

ATTENTION – Reverse days of week / 20 to 1

"Ok, thank you for answering those questions. I just have a few more tasks for you".

UTA ☐

	Participant Response	Correct =no errors	Incorrect	No response	UTA
**1. Can you tell me the days of the week in reverse order, starting with Sunday?**	**Record the order in which the participant responds**<br><br>Sun__Sat___Fri___Thu____Wed___Tues__Mon_<br><br>Comments……………………………………………..	◻	◻	◻	◻
	**Use this line if the first attempt was interrupted - N/A** ◻<br><br>Sun__Sat___Fri___Thu___Wed___Tues___Mon__<br><br>Comments……………………………………………...	◻	◻	◻	◻
**2. Can you count backwards from 20 down to 1?**	**Record the order in which the participant responds**	◻	◻	◻	◻
	20__19__18__17__16__15__14__13__12__11__10__9__8__7__6__5__4__3__2__1__<br><br>Comments………………………………………………………………………………………				
	Use this line if the first attempt was interrupted – N/A ◻<br><br>Record the order in which the participant responds<br><br>20__19__18__17__16__15__14__13__12__11__10__9__8__7__6__5__4__3__2__1__<br><br>Comments………………………………………………………………………………………				

Participant ID – 3 digit no	Site	Date of visit
_______________	A ☐ B ☐ C ☐	____/___/____

ATTENTION– Vigilance A

UTA ☐

Firstly establish a finger tapping method. If no table is close enough, give*n you*r patient you*r clipboard to tap their finger* on. Get them to do it once. If this works well, say:

*"Now I will say some letters and I would like you to tap y*our fingers on this clipboard like before whenever y*ou hear the letter A. We will do a pract*ice *first."*

Read out the following list of letters.

"T A P"

If the participant correctly taps on the letter 'A' then proceed with the task.
If they do not tap, then explain the instructions again and try once more. If they are still unable, move onto the task.
Read out the following list of letters, approximately one letter per second.
Tick box for each letter that patient taps their finger on.

F	B	A	C	M	N	A	A	J	K	L	B	A	F	A	K	D	E	A	A	A	J	A	M	O	F	A	A	B

Circle the appropriate answer -
***No* error (1 point) / One error (1 point) / More than one error (no points)**
Score ________/1 **UTA ☐**

Quick test of long-term memory (Subtest of the DRS-R-98)

PRIORITY
Ask the participant *"Do you remember that I showed you three pictures in the beginning of our meeting? Can I ask you to tell me which of these objects you remember?"*

Correctly identified: **Lemon:** **Yes ☐** **No ☐** **UTA ☐**
Key: **Yes ☐** **No ☐** **UTA ☐**
Ball: **Yes ☐** **No ☐** **UTA ☐**

Other responses:

Participant ID – 3 digit no	Site	Date of visit
_______________	A ☐ B ☐ C ☐	____/___/____

Perceptual disturbances and hallucinations (Subtest of the DRS-R-98)

PRIORITY
"How are you getting on here? Are the staff treating you well? Has anyone been treating you poorly / anyone trying to hurt you?"
"Sometimes people can become afraid of the people or things around them. Have you experienced this?
Do you have beliefs that other people tell you aren't true?"
Indication of delusions: Yes □ No □ UTA □

"Sometimes patients when unwell can develop strange experiences, such as seeing things that other people can't see. Has this happened to you?"
"Has your mind been playing tricks on you so that you're not sure if what you're seeing or hearing is real. Tell me about it?"
"Have you had trouble recognising family or friends. Have they looked or behaved differently?"
Indication of hallucinations: Yes □ No □ UTA □

Comments

Visuospatial Ability (Subtest of the DRS-R-98)

Look around the patient's room/bed space and identify two objects. Ask the patient which object is closer to them.
"Does this room look strange to you in any way?" Ask the participant if the *"room looks tilted"*.
Consider placement of initials and signature on consent form.

Indication of visuospatial disturbance: Yes □ No □ UTA □

Comments

UVA Pain Scale

Ask the participant *"Are you in pain right now?"*: **Yes □ No □ No response □ UTA □**

If yes, how does the participant rate their based on the options from the pain thermometer?
If the participant is unable to score, give your subjective rating.

Score______/10 UTA □ N/A (no pain) □

Participant score □ Assessor □

Delirium Rating Scale-Revised-98

Severity Score (items 1-13) ……… /39

Total Score (items 1-16) ………. /46

Participant ID – 3 digit no	Site	Date of visit
_______________	A ☐ B ☐ C ☐	____/___/____

modified Richmond Agitation Sedation Scale (RASS)

Score ………………

Observational Scale of Arousal (OSLA)

Total score …………………./15

Additional Comment from discussion with participant / relative / staff or case notes

Words/phrases or test scores indicating possible delirium in the past 24 hours<br>*Look for words/phrases like:*<br>• can't hold a conversation<br>• inattentive<br>• seeing things that aren't there<br>• change in mental state<br>• unusual thoughts<br>• slept poorly<br>• disoriented<br>• confusion<br>• incoherent<br>• lethargic<br>• apathetic<br>• drowsy<br>• agitated<br>• muddled<br>• not quite themselves	☐ Yes ☐ No ☐ Not known<br>If Yes (note down the words used):
Comments	

Dementia / Learning disability

Presence of Dementia: Yes ◻ No ◻ Uncertain ◻

Presence of Learning Disability: Yes ◻ No ◻ Uncertain ◻

This is based on the following sources (Tick all that are relevant)
Casenotes ◻ Hospice team ◻ Relative/friend ◻ Dementia drugs ◻
Other ◻ : …………………………………….

Comments

Participant ID – 3 digit no	Site	Date of visit
_______________	A ☐ B ☐ C ☐	____/___/____

Delirium DSM-V delirium diagnostic algorithm for delirium

Criteria A-E Yes □ No □ Unknown □

Presence of Delirium: Yes □ No □ Uncertain □

Note: This is based on DSM-5 diagnostic algorithm for delirium

Group allocation – refer to Grouping Participants SOP for further information

□ Delirium
□ Possible delirium
□ No delirium
□ Undetermined

Reasons for group allocation choice

N.B. Remember to inform the direct clinical care team of the outcome of your assessment

Expert panel review if required for challenging cases (e.g. for 'undetermined' or 'possible delirium')

Document discussion if required

Final designation

□ Delirium
□ Possible delirium
□ No delirium
□ Undetermined

Adverse events

Adverse events were considered by the researcher.
Tick to indicate if any events occurred during the study participation.
AE: **Yes □ No □**
SAE: Yes □ No □
If adverse event, document further in the participant's case notes

Participant ID – 3 digit no	Site	Date of visit
________________	A ☐ B ☐ C ☐	____/____/____

COGNITIVE TESTS
- Days of the week backwards
- 20 to 1
- Vigilance A

Errors on 2 or more tests = inattention ☐

AROUSAL
- RASS – any other than 0 ☐
- OSLA – score >2 ☐

INTERVIEW
- Any behavioural signs that suggest inattention, lack of awareness and orientation such as distractibility, verbal perseverations, etc. ☐

INFORMANT
Evidence of inattention within the last hour ☐

A
A disturbance in attention (i.e. reduced ability to direct, focus, sustain and shift attention) and awareness (reduced orientation to the environment).

→ **NO DELIRIUM**

INFORMANT
Acute onset and/or fluctuation ☐
- Talk to medical team and/or family members
- Check medical notes

B
The disturbance develops over a short period of time (usually hours to a few days), represents a change from baseline attention and awareness, and tends to fluctuate in severity during the course of a day.

→ **NO DELIRIUM**

COGNITIVE TESTS
MEMORY (failure of ANY of the following) ☐
- Recall of 3 words
  - Short/Long term

ORIENTATION
- Time
- Person
- Place

More than 2 of 3 wrong ☐

VISUOSPATIAL
Ability to perceive distance between two object in patient's proximity ☐

INTERVIEW
LANGUAGE ☐
- Assess fluency, grammar, comprehension and semantic content during communication

PERCEPTUAL DISTURBANCE ☐
- Self-report of hallucination and / or delusions

INFORMANT
- Any evidence for cognitive disturbance that is obtained from medical notes or clinical team ☐

C
An additional disturbance in cognition (e.g. memory deficit, disorientation, language, visuospatial ability or perception).

→ **NO DELIRIUM**

INFORMANT
No other pre-existing neurocognitive disorder ☐
- Talk to medical team and/or family members and check medical notes

D
The disturbances in criteria A and C are not better explained by another pre-existing, established, or evolving neurocognitive disorder and do not occur in a context in a severely reduced level of arousal, such as coma.

→ **NO DELIRIUM**

INFORMANT
- Talk to medical team and/or family members and check medical notes to find Evidence for another medical condition as a causal factor ☐

E
There is evidence from the history, physical examination, or laboratory findings that the disturbance is a direct physiological consequence of another medical condition, substance intoxication or withdrawal (i.e. due to a drug abuse or medication), or exposure to a toxin, or is due to multiple etiologies.

→ **NO DELIRIUM**

DELIRIUM DIAGNOSIS CONFIRMED
